# Homologous Recombination and Translesion DNA Synthesis Play Critical Roles on Tolerating DNA Damage Caused by Trace Levels of Hexavalent Chromium

**DOI:** 10.1371/journal.pone.0167503

**Published:** 2016-12-01

**Authors:** Xu Tian, Keyur Patel, John R. Ridpath, Youjun Chen, Yi-Hui Zhou, Dayna Neo, Jean Clement, Minoru Takata, Shunichi Takeda, Julian Sale, Fred A. Wright, James A. Swenberg, Jun Nakamura

**Affiliations:** 1 Department of Environmental Sciences and Engineering, University of North Carolina at Chapel Hill, Chapel Hill, North Carolina; 2 Department of Neurology, UNC Neuroscience center, School of Medicine, University of North Carolina at Chapel Hill, Chapel Hill, North Carolina; 3 Bioinformatics Research Center, North Carolina State University, Raleigh, North Carolina; 4 Department of Biological Sciences, North Carolina State University, Raleigh, North Carolina; 5 Laboratory of DNA Damage Signaling, Department of Late Effects Studies, Radiation Biology Center, Kyoto University, Kyoto, Japan; 6 Department of Radiation Genetics Graduate School of Medicine, Kyoto University, Kyoto, Japan; 7 Medical Research Council Laboratory of Molecular Biology, Cambridge, United Kingdom; 8 Department of Statistics, North Carolina State University, Raleigh, North Carolina; 9 Curriculum in Toxicology, University of North Carolina at Chapel Hill, Chapel Hill, North Carolina; Universitatsklinikum Hamburg-Eppendorf, GERMANY

## Abstract

Contamination of potentially carcinogenic hexavalent chromium (Cr(VI)) in the drinking water is a major public health concern worldwide. However, little information is available regarding the biological effects of a nanomoler amount of Cr(VI). Here, we investigated the genotoxic effects of Cr(VI) at nanomoler levels and their repair pathways. We found that DNA damage response analyzed based on differential toxicity of isogenic cells deficient in various DNA repair proteins is observed after a three-day incubation with K_2_CrO_4_ in REV1-deficient DT40 cells at 19.2 μg/L or higher as well as in TK6 cells deficient in polymerase delta subunit 3 (POLD3) at 9.8 μg/L or higher. The genotoxicity of Cr(VI) decreased ~3000 times when the incubation time was reduced from three days to ten minutes. *TK* mutation rate also significantly decreased from 6 day to 1 day exposure to Cr(VI). The DNA damage response analysis suggest that DNA repair pathways, including the homologous recombination and REV1- and POLD3-mediated error-prone translesion synthesis pathways, are critical for the cells to tolerate to DNA damage caused by trace amount of Cr(VI).

## Introduction

Chromium (Cr) is a naturally occurring element that exists in a variety of oxidation states between -2 to +6. Among these forms, Cr(III) is an essential trace element for normal carbohydrate, lipid and protein metabolism in humans [1]. On the other hand, Cr(VI) has been reported to cause cancer in laboratory animals and occupationally exposed workers [2] and thus draws great attention as a public health concern. Cr(VI) is widely used in various industrial applications including leather tanning, wood preservation, dye production, chrome plating, and alloy manufacturing. Industrial waste containing Cr(VI) may potentially result in the environmental pollution of soil, water and air. Incineration and gasoline usage can also lead to air and water pollution with Cr(VI) as a contaminant. In addition, Cr(VI) can be produced naturally. For example, Cr(III) can be oxidized by Mn(III/IV) into Cr(VI) [2]. Currently, there are hundreds of Superfund sites in the U.S. in which Cr is the major concern for contamination [3].

When taken up by sulfate channels, Cr(VI), which displays no direct DNA damage capability by itself, is reduced by ascorbate, glutathione, and cysteine, producing reactive intermediates of Cr(V), Cr(IV) and reactive oxygen species (ROS) [4]. Cr(III), the final reduction product, can hardly penetrate the cytoplasmic membrane, thus leading to the massive accumulation of Cr inside of cells [2]. The reactive intermediates of Cr has been proposed to induce Cr-DNA adducts and protein-Cr-DNA crosslinks, thereby causing DNA strand breaks and double strand breaks and eventually introducing mutations and genome instability [2]. Besides Cr, ROS can also induce cytotoxicity and mutagenic effects in cells [4]. Due to its genotoxicity, inhaled Cr(VI) was classified as a known human carcinogen by the United States Environmental Protection Agency (U.S. EPA) and the World Health Organization (WHO). With regard to carcinogenicity of Cr(VI) by ingestion, on the other hand, several of the epidemiological studies present contradictory conclusions [5, 6]. In the presence of organic molecules at acidic pH, Cr(VI) can be reduced to non-toxic Cr(III) very quickly, which reduces the concern of Cr(VI) as an ingested human carcinogen. A National Toxicology Program (NTP) conducted a two-year study showing that Cr(VI) is a rodent carcinogen when these rodents were administered extremely high doses (57,300 μg/L or higher) of Cr(VI) in the drinking water [7]. The U.S. EPA has set the maximum contaminant levels (MCL) for total Cr in the human drinking water at 100 μg/L. In California, MCL for Cr(VI) was set at 10 μg/L [8], and Public health goals (PHGs) were established at 0.02 μg/L [9]. Currently the U.S. EPA is re-evaluating these regulations [10]. In 2010, a report by the Environmental Working Group suggests that 89% of the water samples from U.S. cities are contaminated with Cr(VI) at levels ranging from 0.03 to 12.9 μg/L [11]. The mechanism by which Cr(VI) causes genotoxicity is well characterized at concentrations equivalent or higher to current U.S. EPA regulatory levels. However, little information is available regarding the biological effects of Cr(VI) at doses lower than 100 μg/L.

Because of the high level of homologous recombination and the relative ease of gene manipulation, DT40 cells, the chicken B-lymphocyte cells, have been widely used as a model system for higher vertebrate genetic functional studies [12, 13]. With the fast replication speed and the strong phenotypic similarities with murine cells, it makes the DT40 isogenic cell line and its mutants deficient in various genes ideal for reverse genetic studies [14]. Recently, DT40 cells have also been successfully used to measure the genotoxicity of different chemicals [15–17].

Cells are constantly exposed to both endogenous and exogenous agents that may cause DNA damages, of which if not repaired might cause genome instability and induce mutations or cell death. Cells have developed multiple mechanisms to deal with different types of DNA damages[18]. Nucleotide excision repair [19] and Fanconi anemia genes [20] were previously reported to be essential for the removal of Cr(VI)-induced DNA damage or activated in human cells exposed with Cr(VI). Inter-strand DNA cross-links (ICLs) was reported caused by Cr(VI) exposure in the present of glutathione, one report suggests FANCD2, ERCC1 or XPF doesn’t impact cell sensitivity to Cr(VI)[21]. Non-homologous end-joining (NHEJ) repair pathways[22] was reported to be involved into the removal of Cr(VI) induced DNA damaged in Saccharomyces cerevisiae and HR repair pathways were reported to be involved into the removal of Cr(VI) induced DNA damage in mammalian cells[23]. The Werner Syndrome Protein was also reported to be involved into Cr(VI) induced DNA damage[24]. To further investigate the mechanism of Cr(VI) induced DNA damage and repair pathways, in this study we screened a battery of DT40 mutant cells using genotoxicity profiling and found the DNA repair genes and pathways to be critical for cell survival when these cells were exposed to Cr(VI) at nanomolar concentrations. We also confirmed our results using human cancer knockdown cells and further studied the time- and dose- dependent genotoxicity and mutagenicity of nanomolar concentrations of Cr(VI).

## Materials and Methods

### Chemicals and reagents

The following chemicals and reagents were used in the study: potassium chromate (K_2_CrO_4_, Sigma), 2,3-bis[2-methoxy-4-nitro-5-sulfophenyl]-2H-tetrazolium-5-carboxanilide inner salt (XTT) (Sigma), polybrene, puromycin (Sigma), 1-methoxy-5-methylphenazinium methyl sulfate (Sigma), TRIzol^®^ RNA Isolation Reagent (Invitrogen), RPMI 1640 culture medium (Invitrogen), chicken serum (Invitrogen), penicillin/streptomycin (Invitrogen), fetal bovine serum (FBS) (Atlanta Biologicals), iScript^™^ cDNA Synthesis Kit (Bio-Rad Laboratories, Inc.), SsoAdvanced^™^ Universal SYBR^®^ Green Supermix (Bio-Rad), and TransIT^®^-293 transfection reagent (Mirus Bio LLC).

### Cell culture

Chicken DT40 and mutant cells were maintained as described previously [17]. Briefly, DT40 cells were maintained in non-phenol red RPMI 1640 medium with 10% fetal bovine serum, 1% chicken serum and 1% penicillin/streptomycin. The cells were incubated at 39.5°C and 5% CO_2_ with 95% humidity. The source of all DT40 knockout mutant cells is provided in Table 1 in S1 File. Human lymphoblastoid (TK6) cells were kindly provided by Dr. Rebecca Fry (University of North Carolina at Chapel Hill). HeLa and 293T cells were obtained from the Lineberger Comprehensive Cancer Center at the University of North Carolina at Chapel Hill. TK6 cells were maintained in RPMI 1640 medium with 10% fetal bovine serum and 1% penicillin/streptomycin. HeLa and 293T cells were grown in DMEM medium supplemented with L-glutamine, 10% fetal bovine serum and 1% penicillin/streptomycin. All human cells were maintained at 37°C with 5% CO_2_.

### Lentiviral-mediated RNA interference

All short oligonucleotides used for lentiviral plasmids were purchased from Thermo Fisher Scientific Inc. and were inserted into the PLKO.1-puromycin vector. Lentiviral particles were prepared and target cells were infected according to a standard protocol. Briefly, 2μg PLKO.1, 2μg pREV, 2μgpGag/Pol and 1μg pVSVG were transfected with TransIT^®^-293 transfection reagent into HEK293T cells in a 6cm dish. Cell culture medium was changed 24 hours later, and the supernatant containing lentiviral particles was collected 2 days after transfection. Target cells were infected with lentivirus in the presence of 5 μg/ml polybrene, and after 24 hours of incubation fresh medium with puromycin was used. 2–3 days later, resistant cells were used directly (BRCA1 and RAD54) or monoclonal colonies (POLD3) were collected. Knockdown efficiency was checked by qPCR. Details of shRNAs are provided in Table 2 in S1 File.

### Analysis of DT40 cell-based DNA damage response

The DT40 cell-based DNA damage response was analyzed as previously reported [17] with minor modifications. Briefly, DT40 cells were suspended in culture medium (~750 cells per 75 μL per well), seeded into 96-well plates, exposed to K_2_CrO_4_, and allowed to divide for ~7 cell cycles. After cultivation, cell viability was determined by XTT-based cell viability assays.

### *TK* mutation assay

The thymidine kinase locus gene (*TK*) mutation assay was performed to quantify mutagenicity (214074). Background *TK* gene mutants were removed from TK6 cells by growing them for 2 days in media containing CHAT followed by incubation for 1 day in media containing CHT. Cells were then seeded at 2.7×10^5^ cells/ml in 20 ml serum containing media and were exposed to Cr6 for different periods. Following K_2_CrO_4_ incubation, cells were washed three times with sterile PBS, re-suspended in full serum containing media and cultured for 2 days to allow the development of mutations. The cells were maintained at 1.5 × 10^6^ cells/ml or lower to avoid overgrowth. The cells were harvested by centrifugation, re-suspended in fresh media. For the TK-deficient mutant selection, the cells were cloned into 96-well plates at 40,000 cells/well in growth medium (0.125 ml) in the presence of the selective agent trifluorothymidine (TFT, final concentration 4 μg/ml) for the detection of mutation frequency (MF). Cells were also seeded at a density of 1.0 cells/well into 96-well plates in the absence of TFT to determine plating efficiency (PE). The plates were incubated at 37°C in an atmosphere of 5% CO2 for 14 days. The colony formation was then scored. The plates containing TFT were then re-fed with TFT and incubated for an additional 14 days to score for the appearance of slow-growing TK mutants. The total mutation frequencies were calculated according to the Poisson distribution.

### qPCR analysis

Total RNA was isolated using TRIzol^®^ RNA Isolation Reagents, and an equivalent amount of RNA from each sample was used as template to make cDNA using the iScript^™^ cDNA Synthesis Kit. qPCR was performed according to the standard protocol using SsoAdvanced^™^ Universal SYBR^®^ Green Supermix. Primers used in this study were listed in Table 2 in S1 File. Data were analyzed by ΔΔCt method for relative quantifications, actin was used as internal control.

### Statistical analysis

Data are reported as the mean ± standard deviation of at least triplicate samples. Analysis of covariance (ANCOVA) was used to test for mean intercept differences and differences in the slopes of the linear dose-response curves in cell viability analyses between wild-type and a series of mutant cells. For display purposes, survival data were log-transformed, providing approximately normal errors, to calculate the lethal concentration 50 (LC_50_) values for each cell line using Graphpad Prism 5 (La Jolla, CA). *P*-values were two sided and adjusted for multiple comparisons as noted.

To represent the point of departure values for concentration-response, Benchmark dose (BMD) values [25] were obtained from the EPA BMD software v 2.4. These values represent one-standard deviation departures from control values and corresponding lower 5% BMDL values, using log_10_(concentration+0.5) to avoid taking logarithms of zero control values. For each instance, the best fitting result from Hill, exponential and polynomial response non-constant variance models was used. In several instances, the fit was considered marginal by BMD information criteria, and therefore we devised a 4-parameter robust logistic model, fit by maximum likelihood in *R* v3.01, to augment the analyses. In no instance was the BMD value determined from the logistic model outside of the 90% confidence interval reported by the BMD software.

Comparisons of BMD values from independent concentration-response curves were performed using z-statistics computed as $\;z=\left(BMD_{1}-BMD_{2}\right)/\sqrt{SE_{1}^{2}+SE_{2}^{2}},$ with standard errors inferred from the BMD confidence interval. One-sample t-tests were performed for comparisons of LC_50_ across the cell lines in comparison to DT40 wilt-type cells using ratio values and in comparison to a null ratio of 1.0.

## Results

### Chicken DT40 cells deficient in either homologous recombination or the error-prone translesion synthesis pathway are hyper-sensitive to Cr(VI)

**Fig 1:**
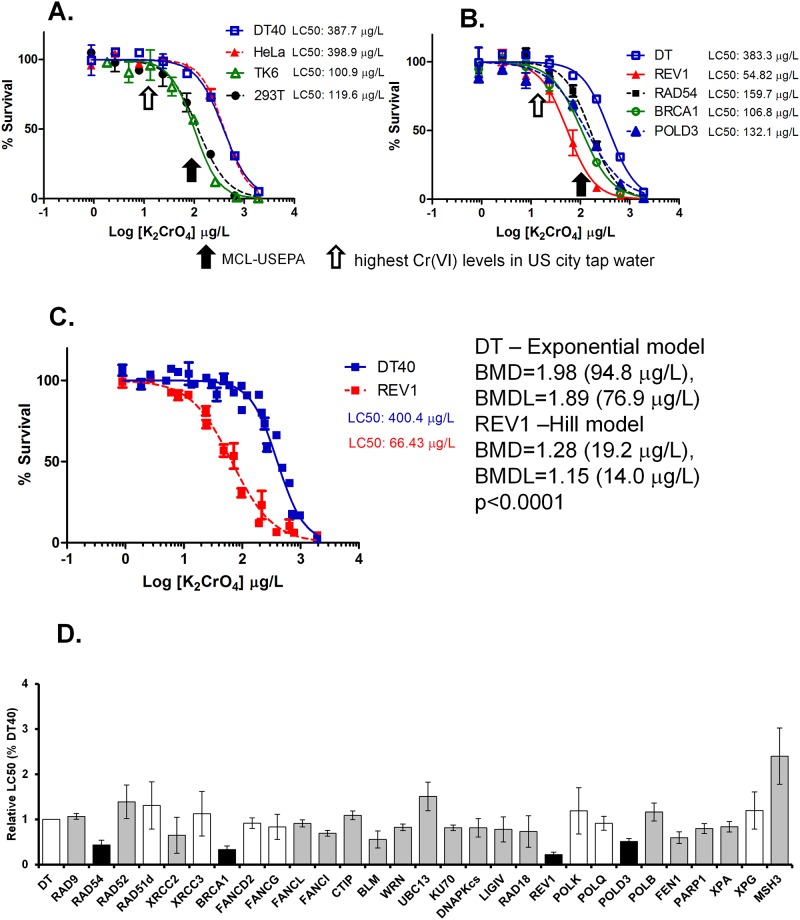
K_2_CrO_4_ reduces viability of human cells as well as DT40 cells deficient in certain DNA repair pathways at the maximum contaminant levels (MCL) set by the U.S. EPA. Various human cells (A) and DT40 parental cells and their mutant cells (B) deficient in *REV1*, *BRCA1*, or *RAD54* were exposed to K_2_CrO_4_ for ~3 days to determine their survival rates. Survival data were log-transformed giving approximate normality using Prism 5. Each LC_50_ value was then calculated. The black arrow indicates the maximum contaminant levels (MCL) set by the U.S. EPA, while the open arrow shows the highest Cr(VI) levels detected in U.S. city water (12.9 μg/L). (C) Point-of-departure analysis showed a significant difference (p<0.0001) in DT40 (log10(BMD) = 1.98 by the Hill model) vs. *REV1*-deficient cells (log10(BMD) = 1.28 (19.2 μg/L) by the Hill model). (D) DT40 cell-based DNA damage response analysis was performed for K_2_CrO_4_ using a series of DT40 mutants. Relative LC_50_ values were normalized according to the LC_50_ value of the parental (wild type) DT40 cells. Error bars represent standard deviation from at least three independent experiments. Student’s t-test was used to test for significance in mean LC_50_ values between DT40 parental cells and each mutant. Columns shaded in gray indicate significant differences (p<0.05) between parental DT40 cells and each mutant. Columns shaded in black show the cell lines (*REV1*, *RAD54*, *POLD3*, and *BRCA1* mutants) that are markedly sensitive to K_2_CrO_4_. (p<0.05 for all four cell lines after Bonferroni adjustment for 32 tests).

To first determine the cytotoxicity of Cr(VI), we exposed chicken DT40 cells, a frequently used cell line for understanding DNA repair gene function studies, with Cr(VI) salt K_2_CrO_4_ at various concentrations ranging from 1 to 2000 μg/L for approximately three consecutive days. We examined cell survival rate as a result of Cr(VI) treatment. The DNA damage response is a very broad term for the network of cellular pathways that sense, signal and repair DNA lesions. In the current study we use cell survival rate as an indication of toxicity and DNA damage response as shown previously [17]. At the dose of 100 μg Cr/L (equivalent to 373 μg K_2_CrO_4_/L), which is the MCL for total Cr in drinking water as set by the U.S. EPA, DT40 cells showed substantial toxicity with ~50% survival rate compared with vehicle-treated cells. Breaking points (departure points from 100% survival rate) were detected at doses ranging between 20–80 μg K_2_CrO_4_/L (Fig 1A).

Cr(VI) exposure appears to lead to Cr-DNA adducts, protein-Cr-DNA crosslinks, or oxidative DNA damage resulting in genotoxicity [2]. Because DNA repair pathways are important for maintenance of genomic integrity, we hypothesized that cells deficient in a certain DNA repair pathway may show altered tolerance to K_2_CrO_4_ treatment relative to wild type cells. To test this hypothesis, a DNA damage response analysis for K_2_CrO_4_ using isogenic DT40 mutant cells was conducted in this study. DNA repair pathways screened in this study include base excision repair (BER), nucleotide excision repair (NER), mis-match repair (MMR), homologous recombination (HR), nonhomologous end joining (NHEJ), translesion DNA synthesis (TLS), Fanconi anemia DNA repair pathway, cell-cycle checkpoint and RecQ helicases. The whole list and function of each gene is shown in Table 1 in S1 File.

Testing these mutant cells allowed us to identify the DNA repair pathways that are critical for cell survival in response to K_2_CrO_4_ exposure. For a quantitative comparison of differential toxicity between each mutants, we calculated the relative LC_50_ (LC_50_ of mutant cells divided by the LC_50_ of the parental DT40 cells). During this screening, we identified four genes, deletion of any one of which led to hypersensitivity of the cells to K_2_CrO_4_ treatment and significantly reduced the relative LC_50_ compared to parental DT40 cells (Fig 1D). These include two genes involved in tanslesion DNA synthesis (TLS), *REV1* (relative LC_50_ = 0.22, *p*<0.05) and *POLD3* (relative LC_50_ = 0.51, *p*<0.05), and two genes involved in homologous recombination, *BRCA1* (relative LC_50_ = 0.33, *p*<0.05) and *RAD54* (relative LC_50_ = 0.44 *p*<0.05) (Fig 1B and 1D). Among these four mutant cell lines, *REV1*-deficient DT40 cells are most sensitive to K_2_CrO_4_ treatment with an LC_50_ at 66 μg/L and a breaking point of cell survival at 19 μg/L (= 5.2 μg Cr/L) compared with LC_50_ at 400 μg/L and breaking point at 95 μg/L (= 25 μg Cr/L) in wild type DT40 cells (*p*<0.0001) (Fig 1C). These results suggest that DNA repair pathways, including the homologous recombination and the error-prone translesion synthesis pathways, are critical for DT40 cells to tolerate genotoxicity induced by Cr(VI). It is also worth noting that *MSH3* deficient cells show hyposensitivity to Cr(VI) exposure (Fig 1D). Beside these hyper- and hypo-snsitivity results, DT40 cell-based DNA damage response analysis demonstrate moderate sensitivity to K_2_CrO_4_ in various mutants including cells deficient in *XRCC2*, *FANCL*, *FANCI*, *BLM*, *WRN*, *KU70*, *DNAPKcs*, *LIGIV*, *RAD18*, *FEN1*, *PARP1*, and *XPA*.

### Cr(VI) exposure leads to genotoxicity in human cells

**Fig 2:**
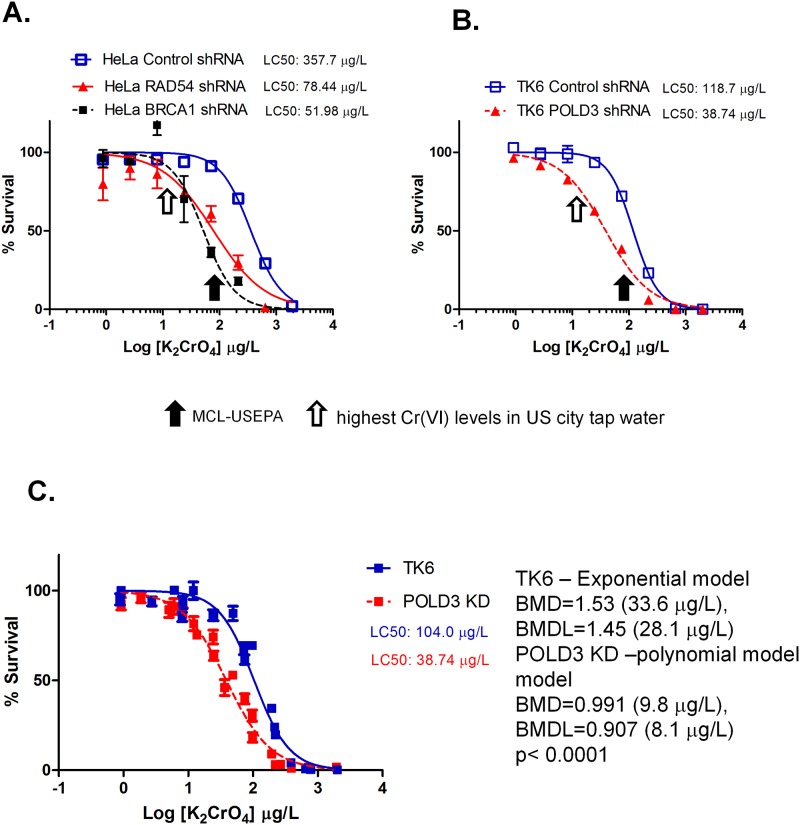
K_2_CrO_4_ reduces the viability of human cells deficient in certain DNA repair pathways at Cr(VI) levels detected in U.S. city water. HeLa cells (A) and TK6 cells (B) transiently or stably knocked down with shRNA against *RAD54*, *BRCA1*, or *POLD3* were exposed to K_2_CrO_4_ for ~3 days to determine their survival rate. (C) Point of departure analysis of TK6 indicated significant differences (p<0.0001) for TK6 control (log10(BMD) = 1.53, exponential model) vs. *POLD3* shRNA knock down (log10(BMD) = 0.99 (9.8 μg/L), polynomial model). The black arrow indicates the maximum contaminant levels (MCL) set by the U.S. EPA. The open arrow shows the highest Cr(VI) levels detected in US city water.

While DT40 cells offer a relatively quick and facile method to analyze the DNA damage response to chemicals, one concern of this approach is that the effects observed in chicken cells may not replicate those in human cells. Therefore, we examined the genotoxicity of human cells induced by Cr(VI). As with DT40 cells, we continuously exposed several different lines of human cells including HeLa (cervical carcinoma epithelial cell line), 293T (kidney epithelial cell line) and TK6 (B-lymphoblast cell line) cells to Cr(VI) salt K_2_CrO_4_ at various concentrations for three days and subsequently assessed cytotoxicity. Similar to DT40 cells, all of the human cell lines tested in this study showed a marked toxic response to Cr(VI) at 373 μg K_2_CrO_4_/L (= 100 μg Cr/L) with survival rate of 50%, 17%, and 10% for HeLa cells, 293T and TK6 cells, respectively) (Fig 1A). We next investigated the DNA damage response of Cr(VI) exposure in human cells deficient in DNA damage repair genes. Since *BRCA1*, *RAD54*, *POLD3* and *REV1* are the genes most critical for chicken DT40 cell survival in the presence of Cr(VI), we investigated whether these genes are also critical for human cell survival. Knockdown of *BRCA1*, *RAD54*, and *POLD3* in human cells was successfully established by lentiviral-mediated RNA interference (Fig 1 in S1 File). However, knockdown of *REV1* led to severe cell death, and we were unable to carry out any further experiments using *REV*1-deficient cells (data not shown). Similar to DT40 mutant cells, human cells deficient in *POLD3*, *RAD54* or *BRCA1* were also more sensitive to Cr(VI) than wild type cells infected with a non-target control virus, as indicated by reduced cell viability (Fig 2). *BRCA1-*deficient HeLa cells showed toxicity with an LC_50_ of 52 μg/L, and *RAD54*-deficient HeLa cells showed toxicity with an LC_50_ of 79 μg/L compared with control HeLa cells with an LC_50_ of 358 μg/L (Fig 2A). *POLD3*-deficient TK6 cells showed toxicity with an LC_50_ of 39 μg/L and a breaking point at 9.8 μg/L (= 2.6 μg Cr/L) compared with control TK6 cells with an LC_50_ of 104 μg/L and a breaking point at 34 μg/L (= 9.0 μg Cr/L) (Fig 2B and 2C). Based on the results from the DT40 mutant cells and human mutant cells, we concluded that trace amount of Cr(VI) have the potential to cause a DNA damage response and genotoxicity during a three day continuous exposure, and homologous recombination (*BRCA1* and *RAD54*) and error-prone translesion synthesis (*REV1* and *POLD3*) are critical for cells to tolerate nanomolar levels of Cr(VI)-induced genotoxicity.

### Cr(VI) exposure-induced genotoxicity is both time and dose dependent

**Fig 3:**
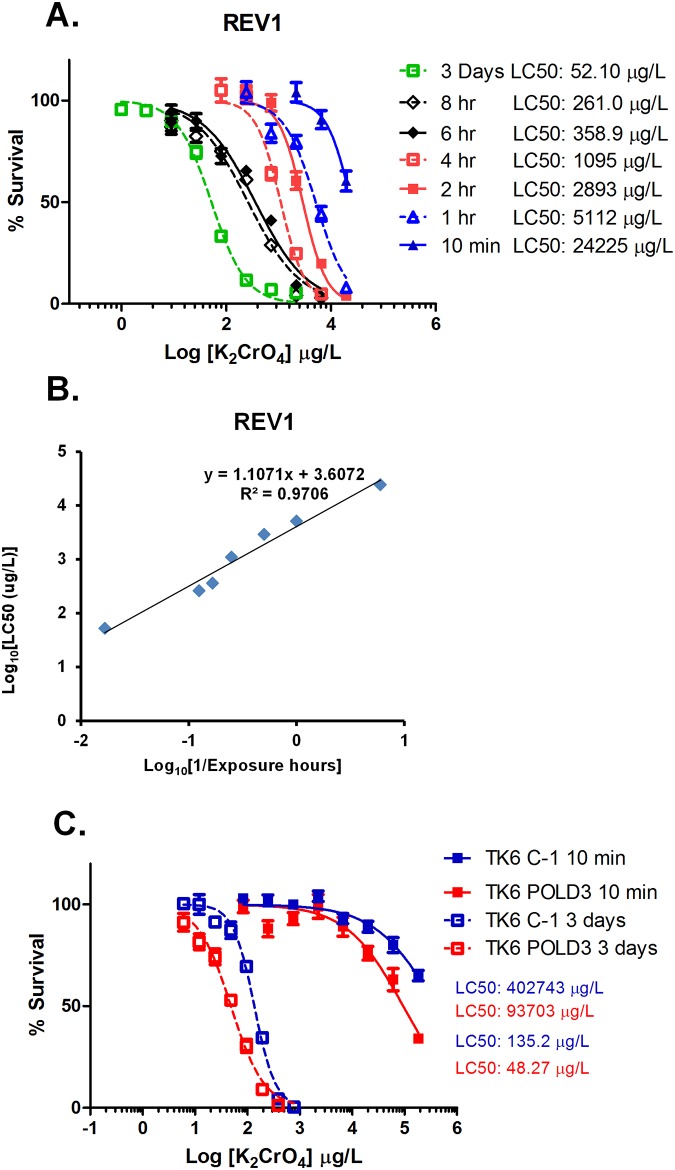
The genotoxicity of K_2_CrO_4_ in human and DT40 cells is drastically decreased when K_2_CrO_4_ incubation time is reduced. (A) *REV1* ko DT40 cells were incubated with K_2_CrO_4_ for 10 min to 8 hours followed by extensive washing. The cells were further cultivated for 3 days in fresh medium without addition of K_2_CrO_4_ to determine cell survival. (B) The exposure times (hours) were multiplicatively inversed followed by log transformation (x-axis). LC_50_ data for each exposure time were log transformed (y-axis). Linear regression analysis was performed to determine the relationship between exposure time and LC_50_ values (p<0.0001). (C) TK6 *POLD3* knock-down cells and TK6 mock shRNA-treated cells were incubated with K_2_CrO_4_ for 10 min followed by extensive washing. The cells were further cultivated for 3 days in fresh medium without addition of K_2_CrO_4_ to determine cell survival. The survival curves were compared between 10-min and 3-day exposure groups (p<0.0001). The green arrow indicates the Cr(VI) concentration (172,000 μg/L) that causes an increase in oral cancer, a finding that was previously shown in an NTP rodent study [7].

It has been reported that efficient uptake of Cr(VI) into cells results in a massive accumulation of intracellular Cr during continuous 24-hour exposure [2, 26, 27]. Therefore, we hypothesized that trace amounts (*i*.*e*. nanomolar levels) of Cr(VI) exposure leads to a DNA damage response only by long-term, and probably not by short-term, incubation of K_2_CrO_4_. To address this possibility, we incubated the most sensitive mutant cells observed in our study, *REV1*-deficient DT40 cells, with K_2_CrO_4_ for durations of 10 min to 8 hours followed by extensive washing of the cells to minimize residual extracellular K_2_CrO_4_ levels. The cells were further incubated for 3 days in fresh media before assessing cell survival rate. *REV1*-deficient DT40 cells showed a drastic time-dependent increase in cell toxicity caused by K_2_CrO_4_ (Fig 3A). A ten-minute K_2_CrO_4_ exposure resulted in cell toxicity with an LC_50_ of ~24 x 10^3^ μg/L, which is 465 times higher (less toxic) than a three-day continuous K_2_CrO_4_ exposure (Fig 3A). We further found a highly significant linear relationship between log-transformed incubation time and LC_50_ values (Fig 3B). Wild type TK6 cells also showed a time dependent toxicity with ~3000-fold higher LC_50_ value upon ten-minute K_2_CrO_4_ exposure than a three-day incubation (Fig 3C). It is worth noting that, under these experimental conditions, wild type and *POLD3*-deficient TK6 cells showed significantly different LC_50_ values (Fig 3C). Upon 10 minute exposure to K_2_CrO_4_, the LC_50_ value is ~403 x 10^3^ μg/L for wild type cells and ~94 x 10^3^ μg/L for *POLD3*-deficient cells. Upon exposure of cells to K_2_CrO_4_ for 3 days, the LC_50_ value is 135 μg/L for wild type cells and 48 μg/L for *POLD3*-deficient cells. Since POLD3 is involved in DNA damage repair, our results suggest that K_2_CrO_4_ treatment leads to a time-dependent increase in induction of DNA damage in human cells.

### The mutagenicity of Cr(VI) in human TK6 cells is both time and dose dependent

**Fig 4:**
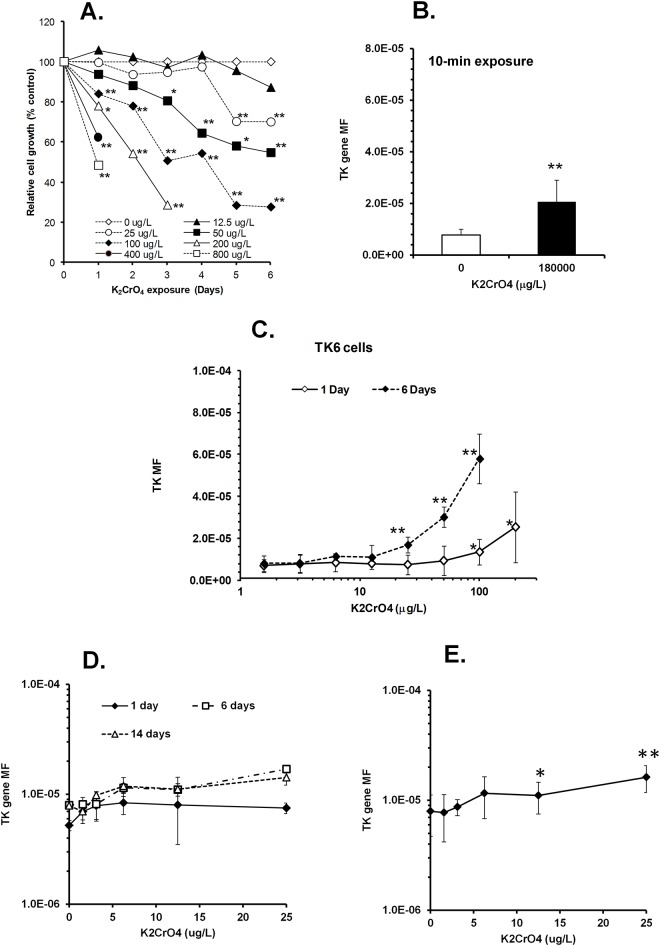
The mutagenicity of K_2_CrO_4_ in human TK6 cells is markedly decreased by reducing K_2_CrO_4_ incubation time. (A) Cumulative cell growth rates of TK6 cells were monitored during continuous incubation with K_2_CrO_4_ at different concentrations for up to 6 days. (B) Mutation rates of TK6 cells exposed to K_2_CrO_4_ for 10 min. After extensive washing, the cells were further cultured for 3 days for the phenotype expression period, followed by a mutant selection process using trifluorothymidine (TFT). The two groups show significantly different mutation rates (p<0.0001). (C) Mutation rates of TK6 cells exposed to K_2_CrO_4_ for 1 or 6 days. For 1-day exposure experiments, the TK6 cells were treated with K_2_CrO_4_ for 24 hours followed by washing and 2-day phenotype expression period before TFT treatment (log10(BMD) = 2.01, Hill model). For 6-day exposure experiments, we continuously treated the cells with K_2_CrO_4_ for 6 days by adding fresh K_2_CrO_4_ into the culture medium when the cells were subcultured. After the 6-day treatment, the cells were treated with TFT (log10(BMD) = 1.48, Hill model). The number of mutants was counted at 2 and 4 weeks after the first TFT treatment. Error bars represent standard deviation around the mean obtained from at least three independent experiments. The 1-day and 6-day BMD values are significantly different (P = 0.005). (D) Dose-response curves of mutation rates of TK6 cells exposed to trace amounts of K_2_CrO_4_ were compared between 1-, 6- or 14-day treatment groups. For 1-day and 6-day exposures, the mutation assays were performed as described in Fig 4C. For 14-day exposure experiments, the assays were conducted as described in the 6-day experiment except that the duration of K_2_CrO_4_ treatment was prolonged. Error bars represent standard deviation around the mean obtained from at least three independent experiments. (E) Since 6- and 14-day dose-response curves overlapped with each other in mutation rates and there was no significant difference between the mutation rates above the second active concentration, the mutation rate data were combined and analyzed as pooled results. (log10(BMD) = 1.02 (10.5 μg/L), Hill model).

Finally, we examined the mutagenic activity of K_2_CrO_4_ and asked whether this activity is time and dose dependent. We exposed human TK6 cells with K_2_CrO_4_ at 180,000 μg/L for 10 min. This concentration of K_2_CrO_4_ is very close to Cr(VI) concentration which has been reported to cause oral and intestine cancers by drinking water in rodents [7]. We found that this K_2_CrO_4_ exposure led to about a two fold increase in the *TK* gene mutation frequency of TK6 cells compared to that of vehicle-treated cells (Fig 4B). A similar increase in TK gene mutation was detected when cells were treated with K_2_CrO_4_ at a concentration between 100 and 200 μg/L for 24 hours (Fig 4C), which is more than three orders of magnitude lower in concentration compared to a 10 min exposure with 180,000 μg/L K_2_CrO_4_ (Fig 4B). We then examined whether prolonged exposure to K_2_CrO_4_ at levels as low as the highest level of contamination found in U.S. city water caused mutations in TK6 cells [11]. Cell growth inhibition in wild-type TK6 cells started at 1, 3, and 5 days when the cells were continuously exposed to K_2_CrO_4_ at 100, 50, and 25 μg/L, respectively (Fig 4A). TK mutation frequency was markedly increased as exposure time increased from 1 day to 6 days with different threshold levels for each exposure condition (Fig 4C). These results clearly show that the genotoxicity introduced by Cr(VI) is dependent on both the concentration of Cr(VI) and the total exposure time. Our data also indicate that short-term Cr(VI) exposure is markedly less genotoxic than chronic exposure at the same Cr(VI) concentration.

When comparing a 6-day K_2_CrO_4_ exposure to a 14-day K_2_CrO_4_ exposure, a drop in the mutation rate observed in the 14-day rate for the lowest active produced an apparent difference in point of departure BMD values (*p*<0.05), however, after eliminating this point there was no evidence of a difference in mutation rates between 6-day and 14-day exposures (Fig 4D). Combining the mutation frequency results from 6- and 14-day K_2_CrO_4_ exposures, a significant increase in mutation rate was observed at 12.5 μg/L (= 6.7 μg Cr(VI)/L) or higher as compared to controls in TK6 cells (Fig 4E). These results suggest that cells may be equipped with a mechanism to tolerate further increases in mutations caused by trace amounts (25 μg/L or lower) of K_2_CrO_4_ for exposure periods of longer than 6 days.

## Discussion

In the current study, we investigated the genotoxicity of low dose Cr(VI) in cultured chicken DT40 cells and human cells. We found that the genotoxicity of cells exposed to nanomolar levels of Cr(VI) is both time and dose dependent, with higher dose or longer exposure leading to severer genotoxicity. We also identified four key components of DNA repair proteins, *REV1*, *POLD3*, *BRCA1* and *RAD54*, that are critical for tolerating the genotoxicity induced by Cr(VI). In addition, we found that prolonged (1–2 weeks) exposure to trace amounts (~10 μg/L) of K_2_CrO_4_ causes increased mutations in human lymphoblastoid cells. However, the genotoxicity of Cr(VI), including the DNA damage response and mutagenesis, decreased ~3000 times when the incubation time was shortened from three days to 10 minutes. Our dose- and time-dependent Cr(VI) genotoxicity results, combined with the quick transit time of Cr(VI) in the digestive tracts of animals after drinking Cr(VI)-contaminated water could explain carcinogenicity of Cr(VI) in drinking water in rodents only at super high concentrations.

### Nucleotide excision repair, but not Fanconi anemia pathway, plays critical roles on the tolerance to DNA lesions caused by K_2_CrO_4_ in DT40 cells

While Cr(VI) is not reactive to DNA under physiological conditions, the intracellular reduction of Cr(VI) towards Cr(III), leading to cause DNA damage[28]. The DNA lesions produced through reduction of Cr(VI) include, single and double strand breaks[29], oxidative DNA damage[28, 30, 31], ternary complex of endogenous reducing agent-Cr-DNA phosphodiester backbone[28, 32], Cr-DNA interstrand crosslinks [33] and DNA-Cr-protein crosslinks [34]. Among various DNA repair pathways for counteracting these DNA lesions, nucleotide excision repair pathway has been reported to be important for the removal or tolerance of Cr(VI)-induced DNA damage [19, 35–37]. In an agreement with these reports, K_2_CrO_4_ caused higher toxicity in DT40 cells deficient in XPA compared to parental DT40 cells.

Fanconi anemia cells are well-known to be hyper-sensitive to DNA interstrand and DNA-protein crosslinking agents such as cisplatin, diepoxybutadiene, and formaldehyde [38, 39]. Previous report showed that FANCA-deficient cells are hyper-sensitive to Cr(VI) in cell survival with S-phase-dependent DSB formation using none-isogenic cell lines and Cr(VI) also induced FANCD2 monoubiquitination [20, 40]. However, other group’s report demonstrated conflicting results using isogenic cultured cells in the presence of physiological levels of ascorbic acid, which is a critical reducing element for Cr(VI)-induced DNA crosslinking lesions *in vitro* [21]. In the DT40 cell-based DNA damage response analysis, we found that the cells deficient in *FANCD2* and *FANCG* were not hypersensitive to K_2_CrO_4_ treatment with marginal sensitivity in cells deficient in *FANCI* and *FANCL*. Our results suggest that DNA crosslinks are not major contributors to the DNA damage response caused by trace amounts of Cr(VI) in the DT40 cell system under the condition we utilized.

### HR and TLS are critical for tolerating Cr(VI) exposure

In our DNA damage response assay using a battery of DT40 cells deficient in DNA repair genes, we found that *REV1*, *BRCA1*, *RAD54*, and *POLD3* are essential in rescuing Cr(VI)-induced DNA damage. This finding suggests that homologous recombination and the error-prone translesion synthesis are critical for DT40 cells to tolerate DNA damage caused by trace levels of Cr(VI). We have confirmed these results using human knockdown cells. Previous reports also demonstrated similar results using Saccharomyces cerevisiae deficient in various DNA repair genes with higher sensitivity of *Rad18 Rad27*, *Rad50*, *Rad51*, *Rad52*, *Rad54*, *Rad59*, *Rev3*, and *Rev1* mutants to either Na_2_CrO_4_ or CrO_3_ [41, 42] compared to their *wild-type* cells. Homologous recombination is one of two primary DSB repair pathways and utilizes a series proteins including ATM, RAD54, and BRCA1 for repairing DSBs during S and G2 phases [18]. The mechanism by which Cr(VI) causes replication-associated DNA DSBs [43] appears to be due to the collapse of replication fork as well as conversion of single strand break to DSBs.

We found DT40 cells deficient in REV1 is the most sensitive cell line to K_2_CrO_4_ among DT40 mutants utilized in this study. As described above, yeast study also showed higher toxicity in the yeasts deficient in RAD18, REV3, and REV1 [41, 42]. In addition, our study demonstrated POLD3-deficient cells were hyper-sensitive to K_2_CrO_4_ with moderate toxicity of RAD18 mutant cells. POLD3 is one of the subunits of replicative DNA polymerase delta and POLD2-POLD3 complex is believed to be involved in TLS by switching binding between POLD1and translesion polymerase REV3 when encountered with DNA replication barrier [44, 45]. Besides, in our preliminary data PCNA mono-ubiquitination deficient mutation cells shows similar Cr(VI) sensitivity with REV1 deficient cells and REV1 C-terminal is critical for REV1’s function to tolerate Cr(VI) toxicity but not REV1 catalytically domain. Taken together, we are proposing the following mechanism of bypassing K_2_CrO_4_-mediated DNA lesions by TLS pathway: 1) ternary Cr-DNA lesions stall DNA replication and activate RAD18-mediated TLS pathway; 2) RAD6-RAD18 complex monoubiquitinates PCNA; 3) low-fidelity TLS polymerase bypass DNA lesions in the presence of scaffold protein REV1, leading to mutations; and 4) REV3 further extends DNA synthesis with POLD2 and POLD3 complex. It is worthwhile to note that previous paper reported polymerase zeta-dependent chromium-induced mutagenesis in yeasts [46]. As with REV3, in the absence of either REV1, or POLD3, we believe Cr(VI)-mediated mutagenesis may be significantly decreased.

## Supporting Information

S1 File This file contains all Supporting Tables (1–2) and Fig 1. Table 1 in S1 File. DT40 mutant cells used in this study. Table 2 in S1 File. Oligonucleotides for shRNA construction and QRT-PCR. Fig 1 in S1 File. Confirmation of knockdown efficiency. BRCA1, RAD54 and POLD3 knockdown cells were prepared and knockdown efficiency were measured by using qPCR. Data are presented as mean ± SD; n = 3.
(PDF)
