# Supplementary material for: Homologous Recombination and Translesion DNA Synthesis Play Critical Roles on Tolerating DNA Damage Caused by Trace Levels of Hexavalent Chromium
Source: PLoS One. 2016 Dec 1;11(12):e0167503. doi: 10.1371/journal.pone.0167503 (PMC5132242; doi:10.1371/journal.pone.0167503)
Supplement: S1 File — Table 1 in S1 File. DT40 mutant cells used in this study. Table 2 in S1 File. Oligonucleotides for shRNA construction and QRT-PCR. Fig 1 in S1 File. Confirmation of knockdown efficiency. BRCA1, RAD54 and POLD3 knockdown cells were prepared and knockdown efficiency were measured by using qPCR. Data are presented as mean ± SD; n = 3. (PDF) [file pone.0167503.s001.pdf]

Supplemental Table 1. DT40 mutant cells used in this study

Gene	Function	References
RAD54	Homologous recombination (HR)	[1]
RAD52	HR	[2]
RAD51c	HR	[3]
RAD51d	HR	[3]
XRCC2	HR, promotion of Rad51 assembly	[3]
XRCC3	HR	[3]
BRCA1	HR, damage checkpoint, transcription-coupled BER, regulation of transcription	[4]
FANCD2	Damage response to interstrand cross-links	[5]
FANCG	Damage response to interstrand cross-links	[6]
FANCL	Damage response to interstrand cross-links	[7]
FANCI	Damage response to interstrand cross-links	[8]
CTIP	DSB repair, DNA resection	[9]
BLM	RecQ helicase responsible for Bloom syndrome	[10]
WRN	RecQ helicase responsible for Werner syndrome	[11]
UBC13	E2 ligase, PRR, HR	[12]
KU70	NHEJ DSB repair	[13]
DNAPKcs	NHEJ DSB repair	[14]
LIGIV	NHEJ DSB repair	[15]
RAD18	Regulation of translesion DNA synthesis (TLS), ubiquitin E3 ligase	[16]
REV1	TLS, deoxycytidyl transferase activity	[17]
POLD3	TLS, subunit of Poldelta	[27]
POLκ	TLS	[18]
POLθ	TLS, base excision repair (BER)	[19]
POLβ	BER	[20]
FEN1	BER, processing of 5' flap during DNA replication	[21]
PARP1	Poly(ADP-ribosyl)ation, BER, repair of DNA SSB and DSB	[22]
XPA	Nucleotide excision repair (NER)	[18]
XPG	NER, transcription-coupled BER	[23]
MSH3	Mismatch repair (MMR)	[24]
ATM	Checkpoint control following double-strand breaks	[25]
RAD9	Cell-cycle checkpoint control as sensors	[26]
RAD17	Cell-cycle checkpoint control as sensors	[26]

Supplemental Table 2. Oligonucleotides for shRNA construction and QRT-PCR

	oligonucleotides	RT-PCR
BRCA1-530		
**Forward sequence:**	5'-CCGGGAGTATGCAAACAGCTATAATCTCGAGATTATAGCTGTTTGCATACTCTTTTTG-3'	5'>ACGGAGCAGAATGGTCAAGTG
**Reverse sequence:**	5'-AATTCAAAAAGAGTATGCAAACAGCTATAATCTCGAGATTATAGCTGTTTGCATACTC-3'	5'>5'CCTGACTGGCATTTGGTTGTA
**RAD54-1324**		
**Forward sequence:**	5'-CCGGCCAGAGTGCAAGCCAGAAATTCTCGAGAATTTCTGGCTTGCACTCTGGTTTTTG-3'	5'>GCCGGTCCTCTCAATAATGTAGC
**Reverse sequence:**	5'-AATTCAAAAACCAGAGTGCAAGCCAGAAATTCTCGAGAATTTCTGGCTTGCACTCTGG-3'	5'>TGGGTCTCACTGCTGGATTTCC
**POLD3-2213**		
**Forward sequence:**	5’CCGGCGAGTCAGCATTGACGATATTCTCGAGAATATCGTCAATGCTGACTCGTTTTTG	5’>CGGGATCCcagagaggaacgaaagggcccc
**Reverse sequence:**	5’AATTCAAAAACGAGTCAGCATTGACGATATTCTCGAGAATATCGTCAATGCTGACTCG	5’>GGGGTACCttatttcctctggaagaagcc

Supplemental Figure 1. Confirmation of knockdown efficiency

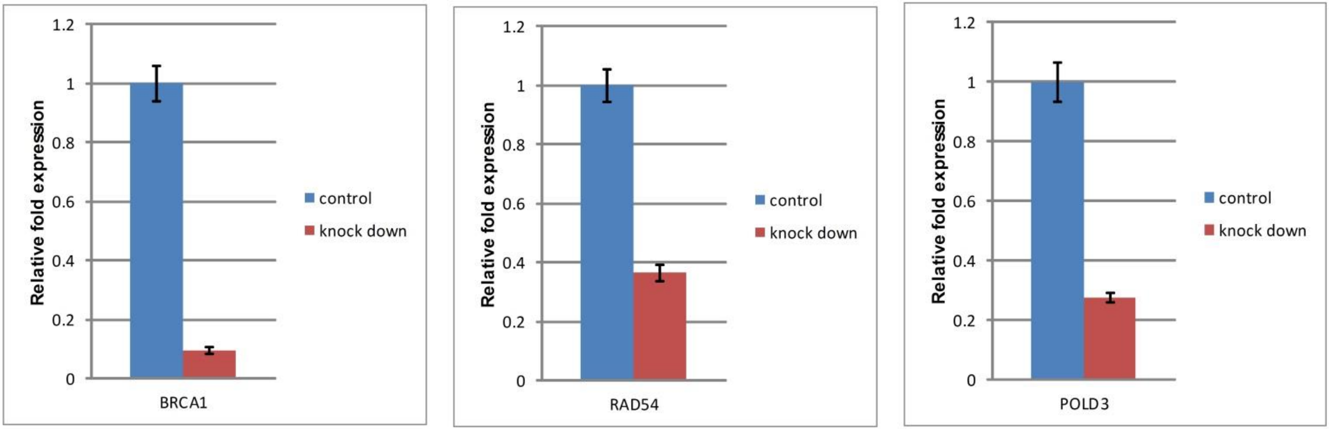

Fig 1. Confirmation of knockdown efficiency. *BRCA1*, *RAD54* and *POLD3* knockdown cells were prepared and knockdown efficiency were measured by using qPCR. Data are presented as mean ± SD; n = 3.

References for Supplementary Information.

1. Bezzubova O, Silbergleit A, Yamaguchi-Iwai Y, Takeda S, Buerstedde JM. Reduced X-ray resistance and homologous recombination frequencies in a Rad54-/- mutant of the chicken DT40 cell line. Cell 89:185–193, 1997.
2. Yamaguchi-Iwai Y, Sonoda E, Buerstedde JM, Bezzubova O, Morrison C, Takata M, Shinohara A, Takeda S. Homologous recombination, but not DNA repair, is reduced in vertebrate cells deficient in RAD52. Mol Cell Biol. 1998 Nov;18(11):6430-5.
3. Takata M, Sasaki MS, Tachiiri S, Fukushima T, Sonoda E, Schild D, et al. Chromosome instability and defective recombinational repair in knockout mutants of the five Rad51 paralogs. Mol Cell Biol 21(8):2858–2866, 2001.
4. Martin RW, Orelli BJ, Yamazoe M, Minn AJ, Takeda S, Bishop DK. RAD51 up-regulation bypasses BRCA1 function and is a common feature of BRCA1-deficient breast tumors. Cancer Res. 67:9658-9665, 2007.
5. Yamamoto K, Hirano S, Ishiai M, Morishima K, Kitao H, Namikoshi K, Kimura M, Matsushita N, Arakawa H, Buerstedde JM, Komatsu K, Thompson LH, Takata M. Fanconi anemia protein FANCD2 promotes immunoglobulin gene conversion and DNA repair through a mechanism related to homologous recombination. Mol Cell Biol. 25:34-43, 2005.
6. Yamamoto K, Ishiai M, Matsushita N, Arakawa H, Lamerdin JE, Buerstedde JM, Tanimoto M, Harada M, Thompson LH, Takata M. Fanconi anemia FANCG protein in mitigating radiation- and enzyme-induced DNA double-strand breaks by homologous recombination in vertebrate cells. Mol Cell Biol. 2003 Aug;23(15):5421-30.
7. Seki S, Ohzeki M, Uchida A, Hirano S, Matsushita N, Kitao H, Oda T, Yamashita T, Kashihara N, Tsubahara A, Takata M, Ishiai M. A requirement of FancL and FancD2 monoubiquitination in DNA repair. Genes Cells. 2007 Mar;12(3):299-310.
8. Sato K, Ishiai M, Toda K, Furukoshi S, Osakabe A, Tachiwana H, Takizawa Y, Kagawa W, Kitao H, Dohmae N, Obuse C, Kimura H, Takata M, Kurumizaka H. Histone chaperone activity of Fanconi anemia proteins, FANCD2 and FANCI, is required for DNA crosslink repair. EMBO J. 2012 Aug 29;31(17):3524-36.
9. Nakamura K, Kogame T, Oshiumi H, Shinohara A, Sumitomo Y, Agama K, Pommier Y, Tsutsui KM, Tsutsui K, Hartsuiker E, Ogi T, Takeda S, Taniguchi Y. Collaborative action of Brca1 and CtIP in elimination of covalent modifications from double-strand breaks to facilitate subsequent break repair. PLoS Genet. 2010 Jan 22;6(1):e1000828.
10. Wang W, Seki M, Narita Y, Sonoda E, Takeda S, Yamada K, Masuko T, Katada T, Enomoto T. Possible association of BLM in decreasing DNA double strand breaks during DNA replication. EMBO J 19:3428–435, 2000.
11. Imamura O, Fujita K, Itoh C, Takeda S, Furuichi Y, Matsumoto T. Werner and Bloom helicases are involved in DNA repair in a complementary fashion. Oncogene 21:954–963, 2002
12. Zhao GY, Sonoda E, Barber LJ, Oka H, Murakawa Y, Yamada K, Ikura T, Wang X, Kobayashi M, Yamamoto K, Boulton SJ, Takeda S. A critical role for the ubiquitin-conjugating enzyme Ubc13 in initiating homologous recombination. Mol Cell. 2007 Mar 9;25(5):663-75.
13. Takata M, Sasaki MS, Sonoda E, Morrison C, Hashimoto M, Utsumi H, et al. Homologous recombination and non-homologous endjoining pathways of DNA double-strand break repair have overlapping roles in the maintenance of chromosomal integrity in vertebrate cells. EMBO J 17:5497–5508, 1998.
14. Fukushima T, Takata M, Morrison C, Araki R, Fujimori A, Abe M, Tatsumi K, Jasin M, Dhar PK, Sonoda E, Chiba T, Takeda S. Genetic analysis of the DNA-dependent protein

kinase reveals an inhibitory role of Ku in late S-G2 phase DNA double-strand break repair. J Biol Chem. 2001 Nov 30;276(48):44413-8.

15. Adachi N, Ishino T, Ishii Y, Takeda S, Koyama H. DNA ligase IV-deficient cells are more resistant to ionizing radiation in the absence of Ku70: implications for DNA double-strand break repair. Proc Natl Acad Sci U S A 98:12109–12113, 2001.
16. Yamashita YM, Okada T, Matsusaka T, Sonoda E, Zhao GY, Araki K, Tateishi S, Yamaizumi M, Takeda S. RAD18 and RAD54 cooperatively contribute to maintenance of genomic stability in vertebrate cells. EMBO J 21:5558–5566, 2002.
17. Simpson LJ, Sale JE. Rev1 is essential for DNA damage tolerance and non-templated immunoglobulin gene mutation in a vertebrate cell line. EMBO J. 22:1654-1664, 2003.
18. Okada T, Sonoda E, Yamashita YM, Koyoshi S, Tateishi S, Yamaizumi M, et al. Involvement of vertebrate Polκ in Rad18- independent postreplication repair of UV damage. J Biol Chem 277:48690–48695, 2002.
19. Yoshimura M, Kohzaki M, Nakamura J, Asagoshi K, Sonoda E, Hou E, Prasad R, Wilson SH, Tano K, Yasui A, Lan L, Seki M, Wood RD, Arakawa H, Buerstedde JM, Hochegger H, Okada T, Hiraoka M, Takeda S. Vertebrate POLQ and POLbeta cooperate in base excision repair of oxidative DNA damage. Mol Cell. 24:115-125, 2006.
20. Tano K, Nakamura J, Asagoshi K, Arakawa H, Sonoda E, Braithwaite EK, et al. Interplay between DNA polymerases beta and lambda in repair of oxidation DNA damage in chicken DT40 cells. DNA Repair 6:869–875, 2007.
21. Matsuzaki Y, Adachi N, Koyama H. Vertebrate cells lacking FEN-1 endonuclease are viable but hypersensitive to methylating agents and H_2O_2. Nucleic Acids Res 30:3273-3277, 2002.
22. Hochegger H, Dejsuphong D, Fukushima T, Morrison C, Sonoda E, Schreiber V, Zhao GY, Saberi A, Masutani M, Adachi N, Koyama H, de Murcia G, Takeda S. Parp-1 protects homologous recombination from interference by Ku and Ligase IV in vertebrate cells. EMBO J. 25:1305-1314, 2006.
23. Kikuchi K, Taniguchi Y, Hatanaka A, Sonoda E, Hochegger H, Adachi N, et al. Fen-1 facilitates homologous recombination by removing divergent sequences at DNA break ends. Mol Cell Biol 25(16):6948–6955, 2005.
24. Nojima K, Hochegger H, Saberi A, Fukushima T, Kikuchi K, Yoshimura M, Orelli BJ, Bishop DK, Hirano S, Ohzeki M, Ishiai M, Yamamoto K, Takata M, Arakawa H, Buerstedde JM, Yamazoe M, Kawamoto T, Araki K, Takahashi JA, Hashimoto N, Takeda S, Sonoda E. Multiple repair pathways mediate tolerance to chemotherapeutic cross-linking agents in vertebrate cells. Cancer Res. 65:11704-11711, 2005.
25. Takao N, Kato H, Mori R, Morrison C, Sonoda E, Sun X, et al. Disruption of ATM in p53-null cells causes multiple functional abnormalities in cellular response to ionizing radiation. Oncogene 18;7002–7009, 1999.
26. Kobayashi M, Hirano A, Kumano T, Xiang SL, Mihara K, Haseda Y, Matsui O, Shimizu H, Yamamoto K. Critical role for chicken Rad17 and Rad9 in the cellular response to DNA damage and stalled DNA replication. Genes Cells. 9:291-303, 2004.
27. Hirota K, Yoshikiyo K, Guilbaud G, Tsurimoto T, Murai J, Tsuda M, Phillips LG, Narita T, Nishihara K, Kobayashi K, Yamada K, Nakamura J, Pommier Y, Lehmann A, Sale JE, Takeda S. The POLD3 subunit of DNA polymerase δ can promote translesion synthesis independently of DNA polymerase ζ. Nucleic Acids Res. 43:1671-83. 2015.
